# Supplementary material for: The effect of language discordance on the experience of palliative care: A scoping review
Source: PLoS One. 2025 Apr 2;20(4):e0321075. doi: 10.1371/journal.pone.0321075 (PMC11964263; doi:10.1371/journal.pone.0321075)
Supplement: Attachment — Submitted filename: Response to Reviewers FINAL.docx [file pone.0321075.s003.docx]

21 January 2025

Weifeng Han, PhD

Academic Editor
PLOS ONE

**Re: Response to Reviewers**

**PONE-D-24-51369:**

**The Effect of Language Discordance on the Experience of Palliative Care: A scoping review**

Dear Dr. Han,

Thank you for taking the time to consider our manuscript for publication in PLOS ONE, we appreciate the opportunity to make revisions based on your feedback, and the feedback of the other two reviewers.

With respect to the role of the funders, we can confirm that they had no involvement/role in the study and would like to add the suggested statement to our original funding acknowledgement (see underlined sentence below):

Production of this document has been made possible through a financial contribution from Health Canada. The views expressed herein do not necessarily represent the views of Health Canada. The funder had no role in study design, data collection and analysis, decision to publish, or preparation of the manuscript.

We would also like to confirm that we have taken this opportunity to review our submission, formatting, and can confirm that the papers cited as part of the data extraction are correct (and that none have been retracted at the time of this revision).

We were pleased with the first reviewer’s very positive feedback on our manuscript, as well as with the thoughtful comments of the second reviewer. Below, you will see the reviewers’ comments in bold, followed by our responses and location of changes in the revised manuscript. We believe that these revisions have greatly strengthened the manuscript.

1. **What was the rationale for conducting a scoping review rather than another type of knowledge synthesis?**

We have revised the text to present the rationale and justification for conducting a scoping review (versus a systematic review); see Lines 107-111 at the very beginning of the “Methods” section.

Synthesis of the literature was conducted using a scoping approach, as it aligned well with our research goal of exploring and mapping out the current literature. With the goal of identifying and synthesizing the broad, heterogeneous range of study designs and findings, our scoping review will be used to inform future research priorities in language discordance in palliative care [14, 15]. To provide a more structured methodological approach, the Preferred Reporting Items for Systematic reviews and Meta-Analyses extension for Scoping Reviews (PRISMA-ScR) Checklist was used to guide reporting of the scoping review procedures and findings [15].

2. **The information on which authors made decisions on article selection was presented in the Limitations section. A brief description should be placed in the Methods section. How were article selection decisions made? How were differences in decisions between the authors resolved?**

We have added details to the manuscript, as suggested by the reviewer; see Lines 128-134 under ‘Eligibility Criteria’ in the Methods section.

The first author led the search, selection process, and data extraction with the support of a Librarian and the second author. Using the mutually developed search terms, the first author performed initial searches and title reviews, documenting reasons for exclusion. The second author reviewed all decisions; any disagreements were discussed and decisions (to include or exclude) were approved by both. The same process was followed through the abstract reviews. We did not experience any disagreements in decision-making and tended to air on the side of inclusion if we questioned the relevance of an article.

3. **Please add details on the data charting and extraction process. Was this done by one researcher or more? Was the data extraction spreadsheet pilot tested? Please describe that process.**

We agree with this suggestion and have added details related to the initial data items for extraction as well as author roles and the data charting/extraction process; see Lines 151-161 under “Data Charting and Data Items” in the Methods section.

Data charting and extraction was completed using a Microsoft Excel Spreadsheet. This scoping review is part of a much larger project exploring palliative the care experiences of underserved populations, and as such, we initially worked with a pre-determined set or common set of data items for extraction: country; target population; study design, setting, participants, and characteristics; terminology and definition of care received; outcomes studied; and author-defined study limitations and next steps. The first author completed the extraction for the first ten articles (in alphabetical order by first author), and these were fully reviewed by the second author and no changes were made to the extraction. The second author completed extraction of the remaining articles. The first author identified a set of initial codes for summarizing key elements in the extracted information, which were reviewed by the second author. Inconsistences and discrepancies identified were discussed and resolved to the satisfaction of both authors. The data extraction summary included below represents the final collaborative findings of both authors.

Again, we thank you for the opportunity to submit minor revisions to our manuscript. We believe that the revised manuscript addresses the issues raised by the reviewers. We look forward to hearing from you on our revised manuscript and to working with you toward its publication.

Sincerely,

Shawn Dookie, RN(EC) MPH NP-PHC
