# Supplementary material for: The effect of language discordance on the experience of palliative care: A scoping review
Source: PLoS One. 2025 Apr 2;20(4):e0321075. doi: 10.1371/journal.pone.0321075 (PMC11964263; doi:10.1371/journal.pone.0321075)
Supplement: Attachment — Submitted filename: Response to Reviewers Feb 26.docx [file pone.0321075.s004.docx]

26 February 2025

Antony Bayer

Academic Editor
PLOS ONE

**Re: Response to Reviewers**

**PONE-D-24-51369:**

**The Effect of Language Discordance on the Experience of Palliative Care: A scoping review**

Dear Dr Bayer,

Thank you for taking the time to consider our manuscript for publication in PLOS ONE, we appreciate the opportunity to make revisions based on your feedback, and the feedback of the other two reviewers.

We agree with your feedback, and took your advice to amend our abstract, to better reflect that our scoping review was quite broad and widely incorporated international data. You will now find this sentence, on line 36, in the “Design” section of our abstract: **“This scoping review was originally designed to explore Canadian official language minority communities but was broadened to an international search for a more robust dataset.”**

Again, we thank you for the opportunity to submit minor revisions to our manuscript. We believe that the revised manuscript addresses the issues you have raised. We look forward to hearing from you on our revised manuscript and to working with you toward its publication.

Sincerely,

Shawn Dookie, RN(EC) MPH NP-PHC
